# Evidence‐based therapy in hypertrophic scars: An update of a systematic review

**DOI:** 10.1111/wrr.12839

**Published:** 2020-06-15

**Authors:** Sebastian P. Nischwitz, Katharina Rauch, Hanna Luze, Elisabeth Hofmann, Alexander Draschl, Petra Kotzbeck, Lars‐Peter Kamolz

**Affiliations:** ^1^ COREMED – Cooperative Centre for Regenerative Medicine, JOANNEUM RESEARCH Forschungsgesellschaft mbH Graz Austria; ^2^ Division of Plastic, Aesthetic and Reconstructive Surgery, Department of Surgery Medical University of Graz Graz Austria; ^3^ KAGes, The Healthcare Company of Styria Graz Austria; ^4^ Medical University of Graz Graz Austria

## Abstract

Hypertrophic scars are still a major burden for numerous patients, especially after burns. Many treatment options are available; however, no evidence‐based treatment protocol is available with recommendations mostly emerging from experience or lower quality studies. This review serves to discuss the currently available literature. A systematic review was performed and the databases *PubMed* and *Web of Science* were searched for suitable publications. Only original articles in English that dealt with the treatment of hypertrophic scars in living humans were analyzed. Further, studies with a level of evidence lower than 1 as defined by the American Society of Plastic Surgeons were excluded. After duplicate exclusion, 1638 studies were screened. A qualitative assessment yielded 163 articles eligible for evidence grading. Finally nine studies were included. Four of them used intralesional injections, four topical therapeutics and one assessed the efficacy of CO_2_‐laser. Intralesional triamcinolone + fluorouracil injections, and topical pressure and/or silicone therapy revealed significant improvements in terms of scar height, pliability, and pigmentation. This systematic review showed that still few high‐quality studies exist to evaluate therapeutic means and their mechanisms for hypertrophic scars. Among these, most of them assessed the efficacy of intralesional triamcinolone injections with the same treatment protocol. Intralesional injection appears to be the best option for hypertrophic scar treatment. Future studies should focus on a possible optimization of infiltrative therapies, consistent end‐point evaluations, adequate follow‐up periods, and possibly intraindividual treatments.

## 1 INTRODUCTION

Scar formation is the physiological response to trauma and the following wound healing cascade of human tissues. Hypertrophic scars are pathologically deviating phenomena that may occur upon an intrinsically or extrinsically altered wound healing cascade. Hypertrophic scars occur in about 30% to 50% after surgery or trauma.^1^ An even higher prevalence can be seen after burn injuries.^2^, ^3^ These scars lead to symptoms like pain, itchiness, or in severe cases restricted mobility due to loss of elasticity and contracture, besides psychological and cosmetic disturbance. Mechanisms causing hypertrophic scar development are still under debate. Most studies implicate that severe inflammation of the wound affect hypertrophic scar formation.^4^, ^5^

Inflammation is crucial for wound healing and is needed for an adequate defense against pathogens as well as for clearing the wound area of debris. In general, wound healing is highly dynamic and self‐limiting. Dysregulated wound healing, characterized by prolonged or increased inflammation is correlated with an overproduction of immature collagen III in contrast to mature collagen I, resulting in increased tissue fibrosis.^6^, ^7^, ^8^, ^9^ One of the key players in tissue fibrosis, hence collagen production, is transforming growth factor beta (TGF‐β) with its subtypes TGF‐β1 and −2 as profibrotic and TGF‐β3 as antifibrotic isoforms.^10^ TGF‐β is involved in triggering events throughout all phases of wound healing.^11^ During inflammation, TGF‐β acts as a potent chemoattractant for neutrophils and macrophages, it regulates immune cell function, and also contributes to resolution of inflammation. In the proliferative phase, TGF‐β has been reported to promote angiogenesis by stimulating endothelial cell migration, differentiation, and capillary tubule formation. Moreover, fibroblast proliferation, fibroblast trans‐differentiation into myofibroblasts, and ECM production, which show abnormal patterns in hypertrophic scars, are mediated by TGF‐β. In addition, TGF‐β inhibits keratinocyte proliferation and enhances keratinocyte migration, promoting re‐epithelialization. Finally, TGF‐β regulates the balance of ECM synthesis and degradation by tightly controlling the production of ECM components and regulating their rate of degradation in the remodeling phase.^11^ An imbalance of these and other involved cytokines are at least co‐responsible in the formation of pathologic scars. Recent results have further confirmed epidermal Foxn1 as a relevant transcription factor in the expression pattern of TGF‐β subtypes.^12^ The exact pathomechanism, however, is not comprehensively elucidated yet. The to date known findings of the influence of TGF‐β and abnormal proliferation suggest interference in these pathways with promotion of apoptosis or abnormal cells as a possible treatment strategy.^13^, ^14^

These insights in the molecular pathways of tissue fibrosis being highly correlated to the inflammatory response can further be a possible explanation for the much higher prevalence of pathologic scars after thermal trauma, since thermal trauma is accompanied by significant systemic,^15^ and local inflammatory responses.^16^, ^17^

The complexity and partially still elusive mechanism behind pathologic scar formation might be one reason for the multitude of treatment regimens available. Many of those are based on suggestions, assumptions, and experience and do somehow interfere with tissue fibrosis and the above‐mentioned pathways. Silicone gel treatment and intralesional injection of immunomodulatory drugs have recently emerged as the most promising treatment regimens, with high quality, randomized, (placebo‐)controlled studies, still being scarce. The exact mechanism of action of silicone sheeting is still inconclusive; increased temperature,^18^ increased hydration,^19^ polarized electric charge leading to scar shrinking,^20^ and others are being discussed.^21^, ^22^ The mechanisms of immunomodulatory drugs are studied more intensively. Especially intralesional triamcinolone (TAC), a glucocorticoid suppressing the inflammatory response, and verapamil (a calcium channel antagonist reducing the synthesis of extracellular matrix) injections have been investigated with regard to their ability to alter the TGF‐β expression patterns,^23^, ^24^ and to positively influence collagen production.^25^ 5‐Fluorouracil (5‐FU) as another rising substance in scar treatment is a pyrimidine anaologue modulating the inflammatory response by inhibiting cell growth, and inducing apoptosis and G2 cell‐cycle arrest among others.^26^, ^27^ Another novel point of action is the hormone angiotensin II: It has been shown to have profibrotic effects,^28^ hence an inhibition of angiotensin‐converting‐enzyme (ACE) represents a reasonable strategy that has been shown effective in animals.^29^

Despite recent advances in the understanding of those substances' mechanisms, studies were still not ultimately able to develop a clinically relevant and effective treatment protocol. One reason for this shortcoming is not least the current lack of an ideal (animal) model for hypertrophic scars,^30^, ^31^ which is needed for thorough understanding of the pathomechanism and potential working points.^32^ Even though advances could be achieved in the red Duroc pig model, the transferability to human scarring remains uncertain.^33^ Another important aspect hampering the advances in scar research is the different phenotype, in which hypertrophic scars can occur. After surgery they appear rather localized and in single strands yielding them suitable for targeted injection therapy, while they appear rather diffuse and heterogenic after burns or secondary wound closure, yielding them more suitable for widespread topical treatment with the possibility of injection therapy in single localized strands. These different occurrences as well as the different stages of ripeness of scars, that also influence the choice of therapy have resulted in standardized human studies having been performed insufficiently.

Mustoe et al suggest “*a move to a more evidence‐based approach in scar management*”, already in 2002.^34^ Ogawa in 2010^35^ and Kafka et al in 2017^36^ conclude similarly that there is an urgent need for high‐quality trials to properly evaluate the effectiveness of the treatment modalities available. Additionally, the nonstandardized evaluation methods and study protocols in between different trials, have, to the authors' knowledge, so far prevented researchers from meta‐analyzing the available studies.

This review has been conducted to investigate the current state of available high‐quality studies for the treatment of hypertrophic scars. The used classification of evidence is the one published by the American Society of Plastic Surgeons (ASPS)^37^; high quality was further defined as a cohort of at least 15 scars per treatment arm (a), a follow‐up of at least 12 weeks after start of the therapy (b), a dropout rate below 20% after 12 weeks (c), and an adequate control group.

This review shall serve as an update to again emphasize the critical need for evidence for this considerable condition.

## 2 METHODS

A systematic review of the literature has been conducted. The methods resembled those used in this previous study,^36^ which have been approved and registered on the International Prospective Register of Systematic Reviews (PROSPERO) under the protocol number CRD42015027040.^38^

### 2.1 Literature sources and search

The online databases *Web of Science* and *PubMed* have been used to review the medical literature covering treatment modalities of hypertrophic scars. The day of accessing the databases was 28 March 2019. Only literature of the past 10 years has been analyzed. The following search term has been used in both databases: [“hypertrophic” AND “scar” AND (“treatment” OR “therapy” OR “scar revision” OR “pressure garment”)]. To minimize the risk of missing relevant data, the MeSH‐term [“Cicatrix, Hypertrophic/therapy”[Mesh]] has been used, additionally.

### 2.2 Reference selection and inclusion criteria

All search results have been listed in an Excel Sheet (Microsoft Excel 2016 MSO [16.0.4849.1000] 32‐bit). In a first step, duplicates have been removed. Second, titles, abstracts and if not unambiguous, full‐text articles have been analyzed concerning the exact study purpose. Again, the same exclusion criteria have been applied as in Reference 36, with the following exclusion reasons: 0 = non‐English, 1 = wrong topic, 2 = in vitro/ex vivo study, 3 = animal study, 4 = keloid study, 5 = review article, 6 = prevention of hypertrophic scars.

Finally, in a third step, the appropriate articles have been analyzed for their scientific value, since only level I evidence articles were to be included. For this assessment, the evidence rating scales of the American Society of Plastic Surgeons (ASPS) have been used,^37^ with the additional refinement of only deeming a study the level of evidence I (“high‐quality, multi‐centered or single‐centered, randomized controlled trial with adequate power”), if the study protocol (a) included a cohort of at least 15 scars per treatment arm, (b) there was a follow‐up of at least 12 weeks after start of the therapy, (c) the dropout rate was below 20% after 12 weeks, and (d) the control group consisted of none, a placebo, or an intervention that is recommended “without restriction” in the most current guideline for the treatment of hypertrophic scars by the working group of scientific medical societies e.V. (AWMF; 4).^39^

The remaining studies were included and have been analyzed for their specific content.

## 3 RESULTS

### 3.1 Quantitative search results

The initial literature search yielded a total of 2672 studies with 1572 results in *PubMed* and 1100 results in *Web of Science*. After duplicate exclusion (1034 duplicates), the remaining 1638 studies were categorized according to the prior mentioned exclusion reasons (0 = non‐English, 1 = wrong topic, 2 = in vitro/ex vivo study, 3 = animal study, 4 = keloid study, 5 = review article, 6 = prevention of hypertrophic scars). The hereby excluded studies number 94 (0), 457 (1), 258 (2), 159 (3), 106 (4), 286 (5), and 115 (6), respectively. The 163 articles meeting the inclusion criteria were then ranked according to their level of evidence: 25, 46, 41, 42, 9 studies for level of evidence V, IV, III, II, I, respectively.

**FIGURE 1:**
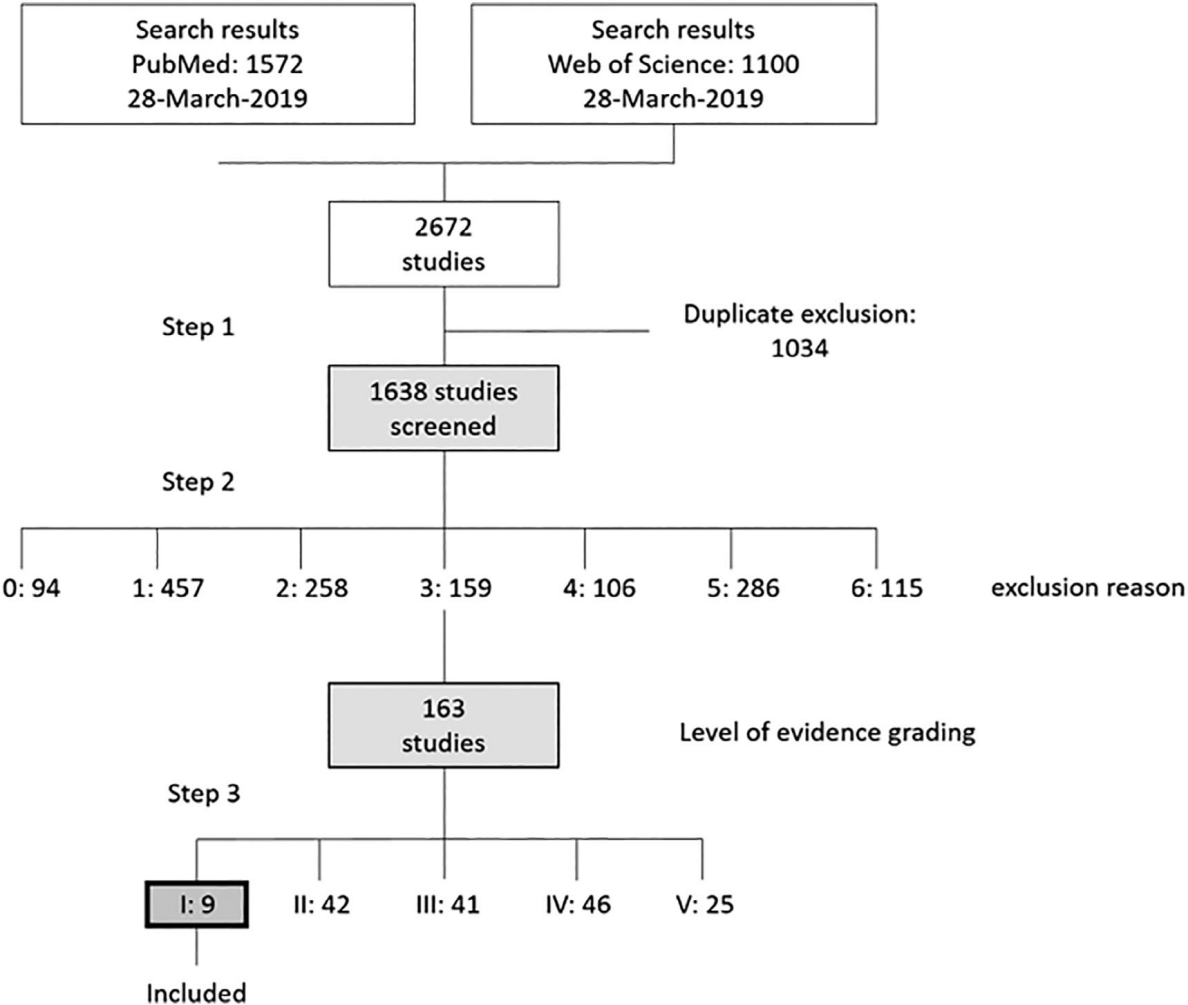
Review process depicted in a flowchart. Exclusion reasons: 0 = non‐English, 1 = wrong topic, 2 = in vitro/ex vivo study, 3 = animal study, 4 = keloid study, 5 = review article, 6 = prevention of hypertrophic scars

Finally, nine studies were included in the qualitative analysis. A flowchart, summarizing the systematic algorithm, as well as the quantitative results can be found in the diagram in Figure 1.

### 3.2 Qualitative search results

A total of nine studies were found suitable for this analysis. Of these nine studies, four compared the effect of intralesional injections, whereof three analyzed triamcinolone (TAC) injection vs intralesional TAC + 5‐FU^40^, ^41^, ^42^ and one investigated the intralesional injection of TAC opposed to that of verapamil.^43^ Two studies investigated the effect of topical ointments (silicone^44^ and enalapril^45^), one that of short‐term massage,^46^ one that of topical silicone dressing, that of pressure therapy and a combination thereof.^47^ The ninth study protocol investigated the effect of CO_2_‐laser on hypertrophic scars.^48^

While the study by Nedelec et al investigating the effect of short‐term massage (5 minutes for three times a week) was the only one yielding no significantly different results,^46^ the other studies could achieve significant improvements in one group over the other(s) in some investigated scar properties.

Study summaries are given in Table 1, while Table 2 summarizes an overview of the significant results.

**TABLE 1:** Summary of the analyzed studies

Study	Treatment	Cohort	Treatment period	Follow‐up	Dropout	Scar entity	End points
Khalid et al^40^	TAC (1x/week 10 mg) vs TAC + 5‐FU (1×/week 4 mg + 45 mg)	120 (:2 groups)	8 weeks	4 weeks	10%	Keloid + Hypertrophic scar (etiology not indicated)	POSAS, height
Khan et al^41^	TAC (1×/week 10 mg) vs TAC + 5‐FU (1×/week 4 mg + 45 mg)	150 (:2 groups)	8 weeks	4 weeks	Not indicated	Keloid + Hypertrophic scar (etiology not indicated)	POSAS, height
Ali et al^42^	TAC (1×/week 10 mg) vs TAC + 5‐FU (1×/week 4 mg + 45 mg)	62 (:2 groups)	8 weeks	4 weeks	Not indicated	Keloid + hypertrophic scar (etiology not indicated)	Height
Abedini et al^43^	TAC (40 mg/mL) vs Verapamil (0.5 mg/cm)	50 (intraindividual)	18 weeks	12 weeks	6%	Keloid + hypertrophic scar (etiology not indicated)	VSS, photograph, pain
Momeni et al^44^	Silicone gel vs Placebo	38 (intraindividual)	16 weeks	16 weeks	10,52%	Hypertrophic burn scar	VSS (excluding height)
Mohammadi et al^45^	Enalapril ointment vs Placebo	30 (intraindividual)	6 months (2× daily)	None	not indicated	Hypertrophic burn scar	Size, thickness, itching score
Nedelec et al^46^	Lotion vs Lotion plus massage (5 min)	70 (intraindividual)	12 weeks (3 times a week)	None	14.3%	Hypertrophic burn scar	Elasticity, skin erythema, melanin, skin thickness
Li‐Tsang et al^47^	Silicone gel dressing vs pressure therapy vs Both vs None	104 (:4 groups)	6 months	1 month	19.23%	Post‐traumatic hypertrophic scar	Color, thickness, pain
Blome‐Eberwein et al^48^	CO2‐laser vs None	48 vs 32 in 36 patients	12‐18 weeks	4–6 weeks	11.11%	Hypertrophic burn scar or post other skin loss diseases	VSS, elasticity, sensation, skin thickness, color, photograph, POSAS

Abbreviations: 5‐FU, fluorouracil; POSAS, Patient and Observer Scar Assessment Scale; TAC, triamcinolone; VSS, Vancouver Scar Scale.

**TABLE 2:** Summary of significant results

Study	Scar height	Scar size	POSAS	VSS	Pliability	Vascularity	Pigmentation	Itching/pain	Recurrence	Complications	
Khalid et al^40^	TAC + 5‐FU > TAC (only significant for keloids)		TAC + 5‐FU > TAC						TAC + 5‐FU < TAC	TAC + 5‐FU < TAC	
Khan et al^41^	TAC + 5‐FU > TAC									TAC + 5‐FU < TAC	
Ali et al^42^	TAC + 5‐FU > TAC										
Abedini et al^43^	TAC > Verapamil			TAC > Verapamil	TAC > Verapamil	TAC	TAC				
Momeni et al^44^				Silicone gel > Placebo							
Mohammadi et al^45^		Enalapril ointment > Placebo						Enalapril ointment > Placebo			
Nedelec et al^46^	Massage = Control				Massage = Control						
Li‐Tsang et al^47^	Combined > Pressure therapy > Silicone gel sheeting > No therapy				All groups		All groups (except redness)	Combined, Silicone gel sheeting > No therapy			
Blome‐Eberwein et al^48^	CO2‐laser > None			CO2‐laser > None			CO2‐laser > None			

Abbreviations: 5‐FU, fluorouracil; POSAS, Patient and Observer Scar Assessment Scale; TAC, triamcinolone; VSS, Vancouver Scar Scale.

## 4 DISCUSSION

The 9 analyzed studies are being discussed in groups differentiating the type of intervention: (a) intralesional injection (TAC, 5‐FU, Verapamil), (b) topical therapy (massage and topical ointments), and (c) CO_2_‐laser.

### 4.1 Intralesional Injection

Four of the nine analyzed studies investigated the effect of intralesional injection in hypertrophic scars. Interestingly, three of them (33% of all level‐of‐evidence‐I‐graded studies) chose to follow the same treatment protocol. While the first^41^ and third^40^ published studies used the Patient and Observer Scar Assessment Scale (POSAS),^49^ and the scar height as primary end points, the second study by Ali et al focused on the scar height, exclusively.^42^ They all used an injection of 10 mg TAC once a week for 8 weeks in one group and a combination of 4 mg TAC and 45 mg 5‐FU once a week for 8 weeks in the other group. The follow‐up of these studies has been chosen quite short with an observation period of only 4 weeks after the last injection (12 weeks after the first injection), while scar remodeling goes on for over 1 year.^50^ Nevertheless, the results yielded significant reduction of POSAS^40^, ^41^ and height^40^, ^41^, ^42^ in both groups, with the 5‐FU group being superior to the TAC‐alone group. The study by Khalid et al^40^ could show no significant difference in between the two study groups when focusing on hypertrophic scars alone, since they did not differentiate between keloids and hypertrophic scars in the inclusion criteria, with the number of hypertrophic scars included being rather low. They also observed a lower recurrence rate in the 5‐FU + TAC group than in the TAC‐alone group, the rate of side effects was also lower (35.2% vs 14.0%).

Another relevant aspect in these studies is the fact, that they acquired large cohorts (150,^41^ 62,^42^ 120^40^), but no intraindividual approach.

The fourth study in this group compared the effect of intralesional TAC (40 mg/mL) injection to that of Verapamil (0.5 mg/cm) in an intraindividual approach with one injection every 3 weeks for a total of 18 weeks; the follow‐up was 12 weeks .^43^ Their primary end points were the Vancouver Scar Scale (VSS), a visual evaluation of a photograph and pain. This group was able to show a superiority of TAC over intralesional Verapamil. Since this study used another dosage/treatment regimen, as well as other validation tools, as the other studies, a direct comparison is not expedient.

In general, the results initially published by Khan et al^41^ could be confirmed by two other studies, leading to the validation of prior conclusion with the combination of intralesional 5‐FU and TAC being a promising treatment approach for hypertrophic scars. TAC significantly suppressed cell proliferation and TGF‐β1 expression, and 5‐FU mainly induced apoptosis, leading to a significant induction of matrix metalloproteinase‐2 and a down‐regulation of production of type I collagen.^26^ The lower effect of TAC on apoptosis might be an explanation for the higher recurrence rates in hypertrophic scars treated with TAC alone. The combination therapy shows a synergistic effect, while reducing the drug dosage, hence also reducing the occurrence of side effects.^23^, ^26^ Future studies should focus on different treatment regimen with different dosage and treatment intervals. In between the studies, it is also recommended to focus on similar/same end points. Also longer follow‐up and intraindividual protocols are recommended. Another possible target substance, which has shown similar regulating mechanisms, is botulinum toxin type A.^51^ Its theoretical mechanism of action has been shown to be in an inhibition of TGF‐b1 as well as in an increase of the JNK phosphorylation leading to reduced fibroblast proliferation and production of profibrotic factors, among others.^52^ No level of evidence I studies have investigated this substance to date.

### 4.2 Topical therapy

Three of the analyzed studies used intraindividual approaches to determine the effect of (a) short‐term massage (+ lotion) vs. lotion alone in 70 patients,^46^ (b) enalapril (ACE inhibitor) ointment vs. placebo in 30 patients,^45^ and (c) silicone gel vs. placebo in 38 patients.^44^ While the short‐term massage (5 minutes, 3 times a week for 12 weeks) yielded no significant difference in the groups, the Enalapril ointment (twice daily for 6 months) led to smaller scars with lower itching scores, yet no results for the indicatedly measured thickness was given. A major downside of these two studies is the fact, that no prolonged follow‐up after the last intervention was indicated, and in the case of Mohammadi et al, no dropout was mentioned, either.^45^

The third intraindividual study compared topical silicone gel vs placebo for 16 weeks.^44^ In this study, a significant amelioration of pigmentation, vascularity, pliability, and itchiness could be achieved; however, no pain difference was reported. One downside is the fact, that there was, again, no prolonged follow‐up after the treatment. The fourth study investigating topical treatments used four groups to determine the effect of silicone gel dressing and pressure therapy alone, a combination thereof, and no treatment.^47^ The treatment lasted for 6 months and an additional month was used as follow‐up period. A combination of the two means was deemed superior to pressure therapy alone, silicone gel dressing, and no treatment, in this order. Yet, silicone gel dressing was superior when it comes to the validation of pain and pruritus as compared to pressure therapy. Color, thickness, VSS and a Visual Analogue Scale for pain were assessed in this study.

Concluding, topical therapeutics represent a valuable, noninvasive approach to the treatment of hypertrophic scars. While silicone gel (dressings) seem to have their effect primarily on pruritus (and pain), the exact effect of physical approaches and other ointments is still to be validated in high‐quality randomized‐controlled trials, focusing on the same end points.

### 4.3 CO_2_‐Laser

One study used the ablative CO_2_‐laser to treat hypertrophic scars.^48^ Forty‐eight scars in 36 patients were treated with a total of three sessions of CO_2_‐laser every 4 to 6 weeks with an energy of 30 W. Thirty‐two scars in the same patients were used as negative controls. Generally spoken, in this study, a significant decrease in scar thickness, pain sensation, erythema, and pigmentation was observed with a nonsignificant, yet present increase in skin elasticity. The control group also yielded an amelioration in the described properties; yet the amelioration curve was steeper in the laser group. With “worse” start points, however, a randomization bias cannot be excluded in this study. In our opinion, a clear recommendation for the use of CO_2_‐laser for the treatment of hypertrophic scars cannot be drawn from this study.

### 4.4 Limitations

With the nature of this review, there are some limitations that should be mentioned. First of all, only studies published within the last 10 years and in English language were investigated. Second, only the databases *PubMed* and *Web of Science* were searched. This of course leaves the risk of studies being missed in this analysis. With the specific criteria chosen to categorize a study as level of evidence I, it is also possible to have excluded important results. Some of the studies that did not match our inclusion criteria and were therefore not considered in the analysis, but deliver important results concerning hypertrophic scars in animal models or other clinical studies, that should be considered are for example, References 53, 54, 55. Another possibility of (exclusion) bias is a misinterpretation of results and/or inclusion/exclusion criteria (not) mentioned in abstract or full‐text articles.

Limitations coming with the nature of the analyzed studies are summarized in the respective discussion sections.

In general, the main downsides having led to exclusion of different studies are short or no follow‐up, small cohorts, and a control group that was not judged as standard of care.

### 4.5 Outlook

Scar therapy and research are an emerging discipline in medical specialties. This leaves plenty of space for the development of new treatment strategies and respective high‐quality studies. The nature of this review limited the included studies to human in vivo studies of high quality that exclusively discussed the treatment of hypertrophic scars, excluding strategies for the prevention of them. While it is not always possible to take preventative measures, in this last section, we would like to discuss a few interesting treatments dealing with prevention and/or not having been tested in humans yet.

The use of amniotic membrane as skin graft dressing for one has been shown to be correlated with rapid reepithelialization and wound healing.^56^, ^57^ With delayed wound healing being a relevant factor in the occurrence of hypertrophic scars,^58^ amniotic membrane has been shown to reduce the occurrence of hypertrophic scars post‐burn.^59^ A thorough understanding of pathomechanism and mechanisms of action, and well designed, prospective studies could encourage a standardized use of preventative measures after (burn) surgery to prevent/reduce the occurrence of hypertrophic scars.

Finally, TGF‐β1 remains one of the key targets in the treatment of hypertrophic scars.^60^ Accordingly, a specific block of TGF‐β1 by, for example, antioxidants or Shikonin, an active component extracted from Chinese herbs, represent a promising approach against hypertrophic scars. These specific blockings have been proven effective in vitro already.^61^, ^62^ Not only the substances per se, also the way of application is a determining factor. The *stratum corneum* of the epidermis usually constitutes an effective barrier for many substances of large molecule size, being an obstacle for active ingredients to get to their place of action. The use of liposomes could therefore represent a valid strategy to properly deliver active substances, rendering painful injections redundant.^63^ The application of papain, a cysteine protease from the papaya fruit, has shown promising results as active agent against hypertrophic scars in vitro and in an animal model when applied within liposomes.^64^

These represent only a few aspects to consider when looking for *the* best strategy in scar treatment, encouraging clinicians as well as basic researchers to pool their competences and reduce the burden of hypertrophic scars.

## 5 CONCLUSION

According to this review, it can be summarized, that even in 2019 there are still few studies, fulfilling the criteria of being rated as level of evidence 1. The last 10 years produced only nine studies in that category. Further research is not only needed in the clinical field, but also in basic science, investigating the mechanisms behind the treatment regimens, thus explaining the exact interference in the TGF‐β pathway, the induction of apoptosis in pathologic skin cells and collagen deposition. Profound insights herein could identify new targets, and new and better treatment regimens. Interdisciplinary approaches are necessary to bridge the gap between basic science and relevant clinical therapy.

Interestingly, four of the analyzed studies date from the year 2018, carefully suggesting a trend toward more evidence for the treatment of the still substantial hypertrophic scars. With a third of all studies investigating the effect of 5‐FU and TAC as infiltratables, this approach can be awarded as the most effective treatment modality with a combination of cell proliferation inhibition and TGF‐β1 expression on one side (TAC), and induction of apoptosis of pathological cells on the other side (5‐FU). As only one treatment protocol has been evaluated, future studies should focus on determining the best regimen in terms of dosage and frequency of injections. Another interesting aspect is the use of topical therapeutics, which also imply a significant symptom relieve for patients and are worth additional investigations. We refrain from distinctly recommending one treatment modality, as the nature and characteristics of hypertrophic scars are too different for recommendations being deducted from this low number of studies. Furthermore, we recommend to design future studies with a longer follow‐up of preferably 12 months, a clear distinction between hypertrophic scars and keloids and the sub‐appearances thereof, an intraindividual treatment approach, and comparable end points evaluated by objective means to allow meta‐analysis of available treatment modalities.

## CONFLICT OF INTEREST

The authors declare no conflict of interest.

## AUTHOR CONTRIBUTIONS

Conceptualization: Sebastian P. Nischwitz, Lars‐Peter Kamolz; Methodology: Sebastian P. Nischwitz , Katharina Rauch; Investigation: Sebastian P. Nischwitz, Alexander Draschl, Katharina Rauch; Visualization: Hanna Luze; Formal Analysis: Elisabeth Hofmann, Petra Kotzbeck; Writing ‐ Draft Preparation: Sebastian P. Nischwitz, Katharina Rauch, Hanna Luze; Writing ‐ Review & Editing: Elisabeth Hofmann, Petra Kotzbeck, Lars‐Peter Kamolz; Supervision: Petra Kotzbeck, Lars‐Peter Kamolz.
